# Glucocorticoid Signaling Pathway: From Bench to Bedside

**DOI:** 10.3390/ijms241311030

**Published:** 2023-07-03

**Authors:** Nicolas C. Nicolaides, George P. Chrousos

**Affiliations:** 1 Division of Endocrinology, Metabolism and Diabetes, First Department of Pediatrics, National and Kapodistrian University of Athens Medical School, “Aghia Sophia” Children’s Hospital, 11527 Athens, Greece; chrousos@gmail.com; 2 Division of Endocrinology and Metabolism, Center of Clinical, Experimental Surgery and Translational Research, Biomedical Research Foundation of the Academy of Athens, 11527 Athens, Greece; 3 University Research Institute of Maternal and Child Health and Precision Medicine, and UNESCO Chair on Adolescent Health Care, National and Kapodistrian University of Athens Medical School, 11527 Athens, Greece; 4 Department of Molecular Genetics, Function and Therapy, The Cyprus Institute of Neurology and Genetics, Nicosia 1683, Cyprus

## 1. Introduction

Glucocorticoids were named by Hans Hugo Bruno Selye, the modern father of stress concepts, for their important role in glucose metabolism [1]. These adrenal cortex-derived “corticoids” were further shown to regulate not only carbohydrate catabolism but also protein and lipid biosynthesis and metabolism; immune, cardiovascular, and brain function; and the overall maintenance of resting and stress-related homeostasis [1,2]. Selye was also the first to demonstrate that glucocorticoids exert potent anti-inflammatory and/or immunosuppressive effects, suggesting that synthetic glucocorticoids might display beneficial therapeutic effects in patients with acute and chronic inflammatory disorders [1,2]. Indeed, three pioneering scientists—Edward Kendall, Philip Hench, and Tadeus Reichstein—succeeded in the isolation and biosynthesis of cortisone, a synthetic glucocorticoid analog, which was then administered to patients with rheumatoid arthritis, showing improvement in their clinical manifestations. For their outstanding efforts and world-recognized results, Kendall, Hench, and Reichstein were jointly awarded the 1950 Nobel Prize in Physiology or Medicine [3]. The tremendous progress in molecular biology has enabled the identification, isolation, and sequencing of the glucocorticoid receptor, a nuclear receptor that binds natural or synthetic glucocorticoids and mediates their pleiotropic actions at the cellular level [4,5]. Since then, genomic, nongenomic, and mitochondrial glucocorticoid actions, signaling, and target tissue sensitivity remain at the center of translational research, connecting basic and clinical researchers through collaborations throughout the world.

In this Special Issue, we have invited scientists with long expertise in the field of glucocorticoid signaling to submit research articles or up-to-date reviews on several aspects of glucocorticoid homeostasis.

## 2. Lessons Learned from Our Special Issue

In the first submitted research article in our Special Issue, Ryu and collaborators designed and used a cellular sensor system to screen compounds for glucocorticoid effector molecules [6]. The authors screened oil extracts with known stress releasing activity and found that L-limonene and L-menthol bound to and activated the glucocorticoid receptor. Among them, L-limonene was shown to reduce the expression of inflammatory genes but not to increase glucocorticoid receptor-mediated transactivation, indicating that L-limonene might function as a selective glucocorticoid receptor agonist (SEGRA). The authors highlighted the novelty and importance of the development of a new rapid cortisol sensor cell system for the identification of GR effector molecules [6].

Mourtzi, Sertedaki, and Charmandari collected and presented the current evidence supporting the notion that stressors might alter the expression of several genes involved in the stress response through epigenetic modifications [7]. The authors described the anatomical and functional components of the stress system and focused on the role of the mineralocorticoid and glucocorticoid receptors in the stress response. They also presented the molecular events occurring following the activation of glucocorticoid receptors leading to epigenetic alterations. Finally, the authors discussed stress-induced epigenetic modifications in the context of several mental disorders, such as depression, post-traumatic stress disorder (PTSD), and others [7].

Kokkinopoulou and Moutsatsou provided an update on the mitochondrial glucocorticoid receptor and its pleiotropic functions [8]. Since the initial identification of functional glucocorticoid response elements (GREs) in mitochondria by the Sekeris group, a large body of evidence has since supported the theory that mitochondrial glucocorticoid receptors substantially influence the expression of the mitochondrial genome, several mitochondrial functions, and the programmed cell death (apoptosis) of several cell types. Importantly, the phosphorylation status of mitochondrial glucocorticoid receptors has been extensively studied in stress-related disorders, including depression. The authors concluded this review by discussing the emerging role of the mitochondrial glucocorticoid receptors in lung and hepatic inflammation, as well as in thermoregulation [8].

Kino, Burd, and Segars reviewed the ever-increasing evidence of glucocorticoid insensitivity in cells infected with SARS-CoV-2 [9]. Following a detailed description of the structure, and the cell invasion and damaging action mechanisms of the virus, the authors described the interference of SARS-CoV-2 within multiple steps of the glucocorticoid receptor signaling pathway, possibly via protein–protein interactions between SARS-CoV-2 proteins and the glucocorticoid receptor or with the transcriptional co-factors of the latter. Through these molecular mechanisms, the authors explain the beneficial therapeutic effects of dexamethasone, a potent synthetic glucocorticoid analogue, in patients with severe COVID-19 [9].

Langendorf and collaborators studied the molecular mechanisms through which glucocorticoids may cause muscle atrophy as a severe adverse effect [10]. To do this, the authors co-cultured primary human umbilical vein cells (HUVECs) with myoblasts and then exposed both cell types to dexamethasone. The addition of the glucocorticoid resulted in reduced expression of CD31 and decreased VEGF release, indicating that dexamethasone might lead to muscle atrophy, as well as to impaired regeneration of muscles following damage of blood vessels. It is worth mentioning that this is the first study in which human primary myoblasts and HUVECs were co-cultured in different experimental conditions [10].

The epigenetics of the stress response has been identified as a promising research field studying the impact of environmental stressful stimuli on gene expression through chemical alterations that occur in cytosine/guanine (CpG) islands in several stress-related genes. Zannas exposed cultured human fibroblast cells to cortisol levels that mimic the in vivo stress response at different time points and studied DNA methylation at the genome-wide level [11]. The author demonstrated that prolonged exposure of cells to cortisol leads to widespread and cumulative alterations in DNA methylation. In addition, the results of this study showed that cortisol-mediated methylation occurred at DNA regions located close to already methylated CpG islands [11].

Canonica and co-workers performed elegant in vitro and in vivo studies, including cellular differentiation, RNA sequencing, and transgenic mice, which examined in vivo retinal morphology changes depending on the corticosteroid sensitivity of retinal pigment epithelium (RPE) cells [12]. The investigators found that RPE is a corticosteroid-responsive tissue in which the hyperactivation of the mineralocorticoid receptor (MR) might contribute to tissue morphologic and functional alterations similar to human pachychoroid pigment epitheliopathy, including RPE phagocytosis, impaired barrier function, and transport in the RPE cells, as well as an increased inflammatory response. Based on their findings, the MR signaling pathway might be recognized as a potential pharmacologic target [12].

Acquired glucocorticoid resistance represents a major limitation of synthetic glucocorticoids prescribed in patients with chronic inflammatory and malignant disorders. Among several molecular mechanisms underlying glucocorticoid resistance, Sevilla and collaborators discussed the role of mitogen-activated protein kinases (MAPKs) and dual-specific phosphatases (DUSPs) in tissue glucocorticoid insensitivity [13]. Accumulating evidence presented in this review article showed that pathologic conditions with aberrant MAPK signaling activation are characterized by impaired anti-inflammatory actions of glucocorticoids, suggesting that members or regulators of the MAPK pathway might be targets for pharmacologic manipulation [13].

In an updated review, Nicolaides and Charmandari discussed the molecular, cellular, and structural biology of the human glucocorticoid receptor and presented the pathophysiology, clinical features, and endocrinologic evaluation of two rare, mirrored pathologic conditions: primary generalized glucocorticoid resistance (Chrousos syndrome) and hypersensitivity syndromes [14]. They also reviewed a number of recently identified *NR3C1* genetic defects causing Chrousos syndrome and discussed recent advances in the pathogenesis and therapeutic management of primary generalized glucocorticoid hypersensitivity syndrome. Finally, the authors highlighted some interesting findings from studies on tissue glucocorticoid sensitivity using –omics technologies [14].

Vassiliadi et al. reviewed the current scientific literature about the role of the hypothalamic–pituitary–adrenal (HPA) axis in health and in critically ill patients with coronavirus disease 2019 (COVID-19) [15]. Through a comprehensive review of physiologic and endocrine aspects of the stress system, the authors presented the pathophysiologic alterations of the stress response that occurred in critically ill non-COVID-19 and COVID-19 patients. They also provided recent findings from clinical samples of patients with COVID-19 showing increased mRNA levels of GRα and glucocorticoid target genes. The authors speculated that the cortisol stress response is strong but frequently insufficient to prevent an excessive inflammatory reaction leading to death [15].

Butz, Mészáros, and collaborators studied the expression profile and function of glucocorticoid-induced microRNAs (miRNAs) in different tissues and cellular models, including glucocorticoid secreting adenomas, as well as H295R and HeLa cells, following exposure to dexamethasone [16]. The majority of the common glucocorticoid-induced miRNAs, among the three groups of experiments, were shown to influence the Wnt signaling system at different levels, including ligand-binding and signal transduction. The authors concluded that hypercortisolism might lead to such alterations in miRNAs, which, in turn, influence the activity of the Wnt signaling pathway that may contribute to the clinical manifestations of patients with Cushing’s syndrome [16].

Zola, Mejlachowicz, and co-workers used Lewis rats to study central serous chorioretinopathy (CSCR), an ocular side effect that occurs upon administration of synthetic glucocorticoids via all routes but not intraocularly [17]. The authors hypothesized that glucocorticoid-mediated suppression of the hypothalamic–pituitary–adrenal (HPA) axis might explain this phenomenon. To this end, rats received either systemic injections of dexamethasone or saline daily for five days to suppress HPA axis activity. The authors showed an increased ratio of MR/GR and 11β-hsd2/11β-hsd1 in the retinal pigment epithelium and the choroid, compared with the retina, suggesting that the observed MR activation might be implicated in the pathogenesis of CSCR [17].

Using zebrafish as an animal model, Dinarello et al. compared the GR-mediated gene expression profile among different zebrafish lines, including the wild-type, the gr knock-out, and a mutant line harboring a point mutation that prevented the DNA binding of the receptor [18]. They also investigated the role of the zebrafish mineralocorticoid receptor (MR) in regulating GR-mediated transcriptional activity. The investigators demonstrated in vivo that the GR might influence gene expression either through direct binding onto DNA or via interactions with other transcription factors, such as the signal transducer and activator of transcription 3 (STAT3). Independently of the molecular mechanism, MR seems to play a fundamental role in the expression of GR target genes. Finally, the authors highlighted the validity of zebrafish as an in vivo model to study the transcriptional activity of GR [18].

Moreno-Rupérez and collaborators investigated the role of glucocorticoid-induced factors, including KLF15 and REDD1, and histone deacetylase 4 (HDAC4) in skeletal muscle proteolysis in the diaphragm and gastrocnemius muscles in septic rats [19]. The authors showed that the administration of lipopolysaccharide (LPS) in these animal models resulted in increased glucocorticoid signaling through the up-regulation of KLF15 and REDD1, decreased IGF-1 expression, and increased expression of HDAC4 in the gastrocnemius compared with the diaphragm. These results might explain the lower level of sepsis-mediated proteolysis in the respiratory muscles than in the locomotor ones, thereby preventing respiratory failure [19].

## 3. Concluding Remarks

In this Special Issue, several experts in the field of glucocorticoid signaling reported their novel findings from both in vitro and in vivo studies, while a significant number of research groups provided updated reviews on topics of special interest. Since the first use of cortisone in patients with rheumatoid arthritis, glucocorticoid signaling remains at the center of translational research, stimulating basic and clinical scientists to join forces towards fruitful collaborations. We express our gratitude to the authors who contributed substantially to this Special Issue, and we wish the readers a productive reading.
